# Strategies to reduce hospital 30-day risk-standardized mortality rates for patients with acute myocardial infarction: a cross-sectional and longitudinal survey

**DOI:** 10.1186/1471-2261-14-126

**Published:** 2014-09-24

**Authors:** Elizabeth H Bradley, Heather Sipsma, Amanda L Brewster, Harlan M Krumholz, Leslie Curry

**Affiliations:** Department of Health Policy and Management, Yale School of Public Health, 60 College Street, PO Box 208034, New Haven, CT 06520-8034 USA; Robert Wood Johnson Clinical Scholars Program, Department of Medicine, Yale University School of Medicine, New Haven, CT USA; Center for Outcomes Research and Evaluation, Yale-New Haven Hospital, New Haven, CT USA; Section of Cardiovascular Medicine, Department of Medicine, Yale University School of Medicine, New Haven, CT USA

**Keywords:** Hospitals, Management, Quality improvement, Acute myocardial infarction

## Abstract

**Background:**

Survival rates after acute myocardial infarction (AMI) vary markedly across U.S. hospitals. Although substantial efforts have been made to improve hospital performance, we lack contemporary evidence about changes in hospital strategies and features of organizational culture that might contribute to reducing hospital AMI mortality rates. We sought to describe current use of several strategies and features of organizational culture linked to AMI mortality in a national sample of hospitals and examine changes in use between 2010 and 2013.

**Methods:**

We conducted a cross-sectional survey of 543 hospitals (70% response rate) in 2013, and longitudinal analysis of a subsample of 107 hospitals that had responded to a survey in 2010 (67% response rate).

**Results:**

Between 2010 and 2013, the use of many strategies increased, but the use of only two strategies increased significantly: the percentage of hospitals providing regular training to Emergency Medical Service (EMS) providers about AMI care increased from 36% to 71% (P-value < 0.001) and the percentage of hospitals using computerized assisted physician order entry more than doubled (P-value < 0.001). Most, but not all, hospitals reported having environments conducive to communication, coordination and problem solving.

**Conclusions:**

We found few significant changes between 2010 and 2013 in hospital strategies or in key features of organizational culture that have been associated with lower AMI mortality rates. Findings highlight several opportunities to help close remaining performance gaps in AMI mortality among hospitals.

**Electronic supplementary material:**

The online version of this article (doi:10.1186/1471-2261-14-126) contains supplementary material, which is available to authorized users.

## Background

Survival rates after acute myocardial infarction differ markedly across hospitals [1] despite overall reductions in AMI mortality nationally [2]. During 2006–2009, 30-day risk-standardized mortality rates (RSMR) after AMI varied from 13.2% to 18.4% at the 5th and 95th percentiles of hospitals, respectively [3]. With more than 800,000 people in the U.S. hospitalized with myocardial infarctions each year [4], closing the performance gap in hospitals could save many lives.

Previous research has identified hospital strategies that have been associated with lower hospital RSMRs and has highlighted key features of organizational culture that are prominent among hospitals with top performance as measured by RSMR [5–8]. Although the presence of these strategies and features of organizational cultural were somewhat limited in 2010, substantial efforts have been made nationally to improve quality, particularly in the wake of public reporting on 30-day mortality rates after AMI [9]. Nevertheless, we lack contemporary evidence about changes in the use of these strategies and features of organizational culture in hospitals nationally.

Accordingly, we sought to describe current use of several strategies and features of organizational culture in a national sample of hospitals, and examine change between 2010 and 2013. We report data on a set of commonly recommended strategies and characteristics that describe organizational culture and compare the current prevalence to that reported by a subsample of the hospitals for which we had similar data from 2010. Findings can help clinicians, policy makers, and researchers seeking to improve quality of care nationally identify what has improved and where additional gaps in practice may persist and require greater attention.

## Methods

### Study design and sample

We conducted a cross-sectional analysis of 378 hospitals (reflecting a 70% response rate among 543 eligible hospitals surveyed between January and November 2013), and a longitudinal analysis of a subsample of 72 hospitals (reflecting a 67% response rate among the 107 hospitals that had also responded to a survey between April and December 2010). We used the data to examine the current prevalence of commonly recommended hospital strategies used to lower mortality rates among patients with AMI as well as features of organizational culture.

For the 2013 survey, we contacted a random sample of 600 hospitals that were part of the American College of Cardiology’s Acute Coronary Treatment and Intervention Outcomes Network (ACTION) registry and treated at least 12 patients with ST-segment elevation acute myocardial infarction per year [9]. This sample size had been previously determined based on the conservative assumption of a 58% response rate in order to have 80% power to detect a difference in RSMR of 1.0 percentage points between 2 groups of 10% of responding hospitals. We sent a letter of invitation to participate in the study to the chief executive officer at all hospitals. We asked the chief executive officer to identify the person most involved in AMI quality improvement efforts, who we contacted to participate in a Web-based survey. Respondents were instructed to coordinate with other relevant staff to complete a single survey reflecting a hospital-level response. Of the 600 hospitals, 54 had ended their participation in the ACTION registry and 3 additional hospitals had closed, leaving 543 hospitals eligible for the follow-up survey. Of these, 378 hospitals (70%) completed a survey between January and November 2013. Additionally, 72 hospitals among the 378 respondents had been previously surveyed as part of our earlier study from April to December 2010 [5]. Among hospitals previously surveyed (N = 107), 35 were non-respondent at follow up (response rate 67%). The 72 hospitals previously surveyed did not differ statistically from the other respondent hospitals in our full sample with respect to teaching status, number of staffed beds, census region, urban/rural location, ownership type, or multihospital affiliation (all P-values > 0.05). We used the subsample of hospitals to explore changes in hospital strategies and organizational culture characteristics between 2010 and 2013.

### Measures

Hospitals responded to a modified version of the survey previously described [5] (See Additional file 1 for questionnaire). Measures included a set of strategies (e.g., employing quality improvement teams focused on post-hospital mortality, physician and nurse champions, pharmacist rounding) as well as features of organizational culture (e.g., communication and coordination across departments, creative problem solving). We also obtained data on hospital characteristics from the 2010 American Hospital Association (AHA) annual survey. Variables included hospital size (total number of hospital beds), teaching status (Council of Teaching Hospitals (COTH)/has accredited residency program/non-teaching), ownership (for-profit/nonprofit/government) and multihospital affiliation (yes/no). We determined census regions from the U.S. Census Bureau and ascertained area type (urban/suburban/rural) using the 2003 Urban Influence Codes.

### Statistical Analysis

We generated frequencies to describe hospital characteristics among our overall and subsample of hospitals. We compared characteristics of these two samples using chi-square tests. We then generated frequencies to describe the current use of hospital strategies and features of organizational culture among the overall sample of hospitals. Last, we generated frequencies of hospital strategies used among the subsample of hospitals for 2010 and 2013 and compared these differences using McNemar’s chi-square tests. A complete case analysis was conducted because only a low proportion of data was missing. We used a significance threshold of P-value < 0.01 given the multiple comparisons. All analyses were completed with SAS 9.3 (Cary, NC). We obtained Internal Review Board exemption (protocol number 1207010622) for our study; participant consent was waived because no identifying participant information was obtained.

## Results

### Hospital sample

Almost 40% of respondents for which data were available on hospital characteristics (N = 358) were teaching hospitals, 73% had fewer than 399 staffed beds, and 90% were in urban locations (Table 1). The hospitals were distributed among all Census regions. The subsample of 72 hospitals that was surveyed in both 2010 and 2013 was not significantly different from the overall sample in terms of teaching status, number of beds, region, geographic location, ownership type, or multihospital affiliation.

**Table 1:** **Hospital characteristics, weighted by hospital volume**

	Overall: 2013 survey (N = 358) ^1^	Subsample: 2010 and 2013 surveys (N = 72) ^2^	Remaining sample: 2013 survey only (N = 286)	P-value ^3^
**Hospital teaching status**				0.445
Council of teaching	54 (15.2%)	14 (19.7%)	40 (14.0%)	
Hospitals member				
Has accredited residency training	88 (24.7%)	18 (25.4%)	70 (24.6%)	
	214 (60.1%)	39 (54.9%)	175 (61.4%)	
Nonteaching				
**Number of staffed beds**				0.115
< 200 beds	115 (32.3%)	21 (29.6%)	94 (33.0%)	
200–399 beds	143 (40.2%)	24 (33.8%)	119 (41.8%)	
400–599 beds	65 (18.3%)	20 (28.2%)	45 (15.8%)	
600+ beds	33 (9.3%)	6 (8.5%)	27 (9.5%)	
**Census region**				0.795
New England	7 (2.0%)	2 (2.8%)	5 (1.8%)	
Middle Atlantic	30 (8.5%)	5 (7.0%)	25 (8.8%)	
East North Central	68 (19.2%)	11 (15.5%)	57 (20.1%)	
West North Central	34 (9.6%)	5 (7.0%)	29 (10.2%)	
South Atlantic	85 (23.9%)	18 (25.4%)	67 (23.6%)	
East South Central	20 (5.6%)	5 (7.0%)	15 (5.3%)	
West South Central	45 (12.7%)	10 (14.1%)	35 (12.3%)	
Mountain	23 (6.5%)	3 (4.2%)	20 (7.0%)	
Pacific	43 (12.1%)	12 (16.9%)	31 (10.9%)	
**Geographic location**				0.993
Urban	321 (90.4%)	64 (90.1%)	257 (90.5%)	
Suburban	15 (4.2%)	3 (4.2%)	12 (4.2%)	
Rural	19 (5.4%)	4 (5.6%)	15 (5.3%)	
**Ownership type**				0.182
For-profit	65 (18.3%)	18 (25.4%)	47 (16.5%)	
Nonprofit	255 (71.6%)	45 (63.4%)	210 (73.7%)	
Government	36 (10.1%)	8 (11.3%)	28 (9.8%)	
**Multihospital affiliation**				0.913
Yes	254 (71.0%)	50 (70.4%)	204 (71.1%)	
No	104 (29.1%)	21 (29.6%)	83 (28.9%)	

^1^20 hospitals missing all AHA data.

^2^1 hospital missing all AHA data.

^3^P-values derived from independent chi-square tests.

### Current strategies

In terms of strategies pertaining to quality improvement and monitoring, the vast majority of hospitals had quality improvement teams devoted to improving inpatient mortality in patients with AMI, had a designated person or group to review deaths of patients with AMI that occurred during hospitalization, and belonged to a regional effort or consortium to improve AMI care (Table 2). In contrast, less than half of hospitals had a quality improvement team focusing on post-discharge deaths or a review process for deaths that occurred within 30 days of admission. In terms of strategies for pre-hospital and inpatient care, several of the strategies were used by less than half of the hospitals, including meeting at least monthly with Emergency Medical Services (EMS) providers to review AMI care, not cross-training nurses to cover in the catheterization laboratory, and having pharmacists rounding on patient with AMI.

**Table 2:** **Description of current strategies used by sample (N = 378 Hospitals)** ^**1**^

Survey item	N (%)
**Quality Improvement and Monitoring**	
Hospital had a QI team devoted to improving inpatient mortality in patients with AMI	289 (79.8%)
Hospital had QI team for improving post-discharge mortality in patients with AMI	163 (45.3%)
Hospital had a designated person or group to review deaths of patients with AMI that occurred during hospitalization	301 (88.8%)
Hospital had a designated person or group to review deaths of patients with AMI that occurred within 30 days of admission	121 (33.9%)
Hospital had a regular morbidity and mortality conferences (or another educational session) to discuss individual cases of patients with AMI	192 (53.9%)
Hospital was part of a regional effort or consortium of hospitals to improve AMI care	289 (80.5%)
**Strategies for Pre-Hospital and In-Patient Care**	
Hospital provided training to EMS providers about AMI care monthly or quarterly	195 (54.5%)
Clinicians from your hospital met with EMS providers to review the care of patients with AMI	
Yes, about monthly	125 (38.8%)
Other than monthly	197 (61.2%)
Hospital had 1 or more physician or nurse champions focused on improving either inpatient or 30-day mortality in patients with AMI	
Neither physician nor nurse champion	87 (24.3%)
Nurse champion only	17 (4.8%)
Physician champion only	40 (11.2%)
Both physician and nurse champion	214 (59.8%)
On inpatient units, hospital had computerized assisted physician order entry	274 (76.3%)
Non-interventional or interventional cardiologists or cardiology fellows were at the hospital 24-hours/day and 7-days/week	90 (24.7%)
Nurses in at least one of your critical care areas were cross-trained to cover in the catheterization laboratory	52 (14.5%)
Which of the following best describes the role of pharmacists in caring for patients with AMI during this time?	
Pharmacists round on all patients in the CCU or with AMI	162 (48.8%)
Pharmacists do not round, but review the medications of all patients with AMI	102 (30.7%)
Pharmacists do not have a specific role in care of patients with AMI	68 (20.5%)
**Organizational Culture**	
Clinicians are encouraged to creatively solve problems related to AMI care processes.	
Never, rarely, or sometimes	47 (13.1%)
Usually or always	311 (86.9%)
There is good coordination among the different departments involved with the care of patients with AMI.	
Never, rarely, or sometimes	36 (10.1%)
Usually or always	322 (89.9%)
Clinicians caring for patients with AMI share new evidence-based approaches with the AMI team.	
Never, rarely, or sometimes	69 (19.3%)
Usually or always	288 (80.7%)
Departments caring for patients with AMI (e.g., cardiology, emergency medicine) communicate easily with each other.	
Never, rarely, or sometimes	32 (9.0%)
Usually or always	325 (91.0%)
Mistakes have led to positive changes in AMI care processes at the hospital.	
Never, rarely, or sometimes	89 (25.1%)
Usually or always	265 (74.9%)

^1^Number of missing responses ranged generally from 14 to 22, with two items that had 46 and 56 missings, respectively.

Several features of organizational culture were prominent in the full sample of hospitals surveyed in 2013. For instance, 87% of hospitals reported that clinicians were encouraged to creatively solve problems related to AMI care processes, and easy communication among departments caring for patients with AMI was reported by 91% of hospitals (Table 2). In terms of collaboration and coordination with partners outside of the hospital, 39% of hospitals held monthly meetings between clinicians and EMS providers to review the care of AMI patients. Somewhat more hospitals (55%) provided training to Emergency Medical Service (EMS) providers monthly or quarterly.

### Changes in strategies

Between 2010 and 2013, the use of many strategies increased but only three increased significantly; the percentage of hospitals that had a quality improvement team to improve post-discharge mortality in patients with AMI increased from 23.6% to 43.5% (P-value = 0.024), and the percent of hospitals providing training to EMS providers about AMI care at least monthly or quarterly increased from 36.1% to 60.9% (P-value < 0.001). The percentage of hospitals using computerized assisted physician order entry increased from 33.3% to 82.6% (P-value < .001) (Table 3). A couple of features of organizational culture worsened, notably coordination among different departments (4.2% to 16.2% of hospitals reported they never, rarely, or sometimes versus usually or always had good coordination, P-value = 0.012), and communication across departments caring for patients with AMI (5.6% to 17.9% of hospitals reported they never, rarely, or sometimes versus usually or always had good coordination, P-value = 0.035) (Table 3).

**Table 3:** **Description of baseline and follow-up strategies (N = 72 Hospitals)**

Survey Item	Baseline ^1^2010 survey N (%)	Follow up ^2^2013 survey N (%)	McNemar’s P-value
**Quality Improvement and Monitoring**			
Hospital had a QI team devoted to improving inpatient mortality in patients with AMI	45 (62.5%)	54 (77.1%)	0.108
Hospital had QI team to improve post-discharge mortality in patients with AMI	17 (23.6%)	30 (43.5%)	0.024
Hospital had a designated person or group to review deaths of patients with AMI that occurred during hospitalization	63 (87.5%)	58 (84.1%)	0.607
Hospital had a designated person or group to review deaths of patients with AMI that occurred within 30 days of admission	16 (22.2%)	25 (36.2%)	0.163
Hospital had a regular ‘morbidity and mortality’ conferences (or another educational session) for discussing individual cases involving patients with AMI	40 (55.6%)	35 (51.5%)	0.851
Hospital was part of a regional effort or consortium of hospitals to improve AMI care	53 (73.6%)	56 (81.2%)	0.442
**Strategies for Pre-Hospital and In-Patient Care**			
Hospital provided training to EMS providers about AMI care monthly or quarterly	26 (36.1%)	42 (60.9%)	<0.001
Clinicians from your hospital met with EMS providers to review the care of patients with AMI			0.458
Yes, about monthly	29 (40.3%)	32 (51.6%)	
Other than monthly	43 (59.7%)	30 (48.4%)	
Hospital had 1 or more physician or nurse champions focused on improving either inpatient or 30-day mortality in patients with AMI			0.518^3^
Neither physician nor nurse champion	23 (31.9%)	17 (24.6%)	
Nurse champion only	4 (5.6%)	1 (1.5%)	
Physician champion only	9 (12.5%)	11 (15.9%)	
Both physician and nurse champion	36 (50.0%)	40 (58.0%)	
On the inpatient units, hospital had computerized assisted physician order entry	24 (33.3%)	57 (82.6%)	<0.001
Non-interventional or interventional cardiologists or cardiology fellows were at the hospital 24-hours/day and 7-days/week	10 (14.5%)	16 (23.2%)	0.238
Nurses in at least one of your critical care areas were cross-trained to cover in the catheterization laboratory	12 (16.7%)	8 (11.6%)	0.607
Which of the following best describes the role of pharmacists in caring for patients with AMI during this time?			0.915^3^
Pharmacists round on all patients in the CCU or with AMI	32 (45.7%)	31 (46.3%)	
Pharmacists do not round, but review the medications of all patients with AMI	24 (34.3%)	23 (34.3%)	
Pharmacists do not have a specific role in the care of patients with AMI	14 (20.0%)	13 (19.4%)	
**Organizational Culture**			
Clinicians are encouraged to creatively solve problems related to AMI care processes.			1.000
Never, rarely or sometimes	12 (16.7%)	12 (17.7%)	
Usually or always	60 (83.3%)	56 (82.4%)	
There is good coordination among the different departments involved with the care of patients with AMI.			0.012
Never, rarely, or sometimes	3 (4.2%)	11 (16.2%)	
Usually or always	69 (95.8%)	57 (83.8%)	
Clinicians caring for patients with AMI share new evidence-based approaches with the AMI team.			1.000
Never, rarely, or sometimes	14 (19.4%)	12 (17.7%)	
Usually or always	58 (80.6%)	56 (82.4%)	
Departments caring for patients with AMI (e.g., cardiology, emergency medicine) communicate easily with each other.			0.035
Never, rarely or sometimes	4 (5.6%)	12 (17.9%)	
Usually or always	68 (94.4%)	55 (82.1%)	
Mistakes have led to positive changes in AMI care processes at the hospital.			0.557
Never, rarely, or sometimes	19 (26.4%)	23 (33.8%)	
Usually or always	53 (73.6%)	45 (66.2%)	

^1^Number of missing responses range from 0 to 3.

^2^Number of missing items range from 3 to 5; one item missing 10.

^3^Tests of symmetry used.

## Discussion

We observed modest changes in hospital strategies and features of organizational culture for hospitals surveyed in 2010 and 2013. The overall direction in terms of strategies used was toward improvement, although few of the changes were statistically significant. The overall direction of features of organizational culture worsened, with 2 of 5 of the organizational culture indicators being significant.

Several hospital strategies associated with lower AMI RSMR in previous research highlight room for further improvement, with levels of adoption under 60% at baseline and at follow up. These strategies largely pertained to inpatient care including having both physician and nurse champions focused on improving AMI mortality [5, 10], having cardiologists or cardiology fellows on site 24 hours a day, 7 days a week [5, 11, 12] (or among hospitals without such cardiologist coverage, having pharmacists round on all patients with AMI [5]), and having clinicians meet with EMS providers monthly for training and to review AMI care [5, 8, 13]. Notably, these strategies are complex interventions that require coordination among units or organizations, and in some cases require substantial investment by hospitals. One strategy in particular, having cardiologists on site 24/7, is resource intensive and may not be feasible for many hospitals.

We found significant increases in hospitals providing regular training to EMS providers. Previous qualitative studies have suggested multiple avenues exist for engaging EMS providers in AMI care; high-performing hospitals in AMI care have been found to actively engage EMS providers in quality improvement activities, invest in strong communication and coordination with EMS agencies and maintain a high level of respect for EMS as valued professionals and colleagues [6]. Although few studies have focused on hospital interaction with EMS providers, a survey of EMS agencies indicated that greater medical supervision of agencies is associated with stronger cardiovascular care procedures [14]. Given the key contributions that EMS providers make in AMI care [15], greater attention to this aspect of care may be warranted.

Another hospital strategy that significantly increased during our study period was computerized physician order entry (CPOE), which has generally been linked to higher quality care [16] and adherence to best practices in AMI care processes [17]. Unintended negative impacts have also been documented, however, depending on the design and implementation of specific CPOE systems [18, 19]. These have included in the earlier days of CPOE implementation perceived loss of control by clinicians [18] and increases in medication error risk [19]. Nevertheless, the substantial rise in CPOE between 2010 and 2013 is not surprising given the improvement in the systems, adaptation by clinicians, and financial incentives for hospitals and providers to adopt electronic health record systems over that time period [20].

Finally, more hospitals reported having quality improvement teams working on improving 30-day mortality after AMI in the follow up compared with the baseline survey. Such re-focusing requires substantial commitment of resources and acceptance of a larger role for hospitals in the post-discharge quality of care. At the same time, some features of organizational culture conducive to high quality AMI care appeared to be less prevalent in the follow up compared with the baseline survey. In particular, coordination and communication across departments caring for patients with AMI still remained a challenge for a notable minority of hospitals and was reported less commonly at follow up than in the baseline survey.

Our findings should be interpreted in light of several limitations. First, the relatively small size of our longitudinal sample means that we were only able to detect changes of large magnitude. Our data suggest that some additional strategies may have increased over time more modestly; for example, the proportion of hospitals with a quality improvement team focused on post-discharge AMI mortality increased from 24% to 44% but the result was not statistically significant. Second, the time period covered by our study, 2010–2013, was fairly short and may not have been sufficient for hospitals to implement some strategies such as monthly meetings with EMS providers. Third, the sample was drawn from hospitals participating in the ACTION registry and therefore may be more invested in quality improvement for cardiovascular care than other hospitals, and within the sample, respondents may have been those that were focusing greater attention on AMI care processes. Such effects may potentially lead to overestimates of the changes in strategy use; thus the magnitude of changes in the use of strategies examined may be more modest among other hospitals.

## Conclusions

In conclusion, we found an overall trend toward greater adoption of strategies that have been found to be associated with lower 30-day AMI mortalities rates, although most changes were modest in size and non-significant, and cross-departmental communication and coordination remain challenging for a small but notable group of hospitals. Improvements may be due in part to extensive previous efforts within the ACTION registry, from which the sample of hospitals was drawn, to improve quality of AMI care. The findings nonetheless highlight important areas for future improvement. Several evidence-based strategies showed low levels of adoption which, if adopted more widely, could help close remaining performance gaps in AMI mortality among hospitals.

## Electronic supplementary material

Additional file 1: **Survey Instrument**. This pdf file contains the survey questions administered to hospitals in 2010 and 2013. (PDF 140 KB)

## Abbreviations

AMI: Acute myocardial infarction
EMS: Emergency medical service
RSMR: Risk-standardized mortality rate
ACTION: Acute coronary treatment and intervention outcomes network
AHA: American hospital association
COTH: Council of teaching hospitals
CPOE: Computerized physician order entry.
