# Supplementary material for: Strategies to reduce hospital 30-day risk-standardized mortality rates for patients with acute myocardial infarction: a cross-sectional and longitudinal survey
Source: BMC Cardiovasc Disord. 2014 Sep 24;14:126. doi: 10.1186/1471-2261-14-126 (PMC4182840; doi:10.1186/1471-2261-14-126)
Supplement: Supplementary file 1 — Additional file 1: Survey Instrument. This pdf file contains the survey questions administered to hospitals in 2010 and 2013. (PDF 140 KB) [file 12872_2014_772_MOESM1_ESM.pdf]

Note: Please reflect upon your hospital's quality improvement efforts that are **CURRENTLY** in place.

Strategies for Improving Acute Myocardial Infarction (SAMI) INITIATIVE SURVEY

Directions: This survey is being conducted to gather national data on hospital efforts to reduce 30-day mortality rates for patients with acute myocardial infarction (AMI). Please consult your colleagues for answers to questions you may not know yourself, so the survey reflects responses for the hospital. If your hospital is part of a multihospital system, please answer on behalf of your hospital only. All information you provide is completely confidential, electronically secure, and only accessible by researchers at the Yale School of Public Health. We will not disclose the source of information given or report or publish data in a way that identifies you or your organization. Patient-level data are not collected. The survey is located on a secure, confidential website. We appreciate your time. If you need any assistance, please contact marcia.mulligan@yale.edu

1. Name: ____
   Position or title: ____
   Hospital: ____
   Role of any colleagues consulted: ____________________________________

2. Is your hospital a member of a multihospital system? *(If no, please skip to Question 5)*
   O Yes O No

3. What multihospital system does your hospital belong to? ______________________________

4. Counting your own hospital, how many hospitals belong to this multihospital system?
   ____

Part I: Hospital Protocols and Processes for AMI Care

5. Does your hospital monitor its risk-standardized 30-day mortality rate for patients with AMI?
   O Yes O No

6. Does your hospital have a quality improvement team(s) devoted to improving the following?
   a. Inpatient mortality in patients with AMI
   O Yes O No
   b. Post-discharge mortality (death occurring after discharge, but within 30 days of admission) in patients with AMI
   O Yes O No

7. Reducing readmissions is a hospital-wide objective.
   O Yes O No

8. Does your hospital have one or more physician champions focused on reducing 30-day mortality in patients with AMI?
   O Yes O No

Note: Please reflect upon your hospital's quality improvement efforts that are **CURRENTLY** in place.

9. Does your hospital have one or more nurse champions focused on reducing 30-day mortality in patients with AMI?
   O Yes O No

10. Does your hospital have a staff cardiologist on site 24/7 (interventional cardiologist or non-interventional cardiologist)?
   O Yes O No

11. Does your hospital have a cardiology fellow on site 24/7?
   O Yes O No

12. Does your hospital have a designated person or group to review the deaths of patients with AMI (i.e., on an individual case level) that occurred during:
   a. hospitalization?
   O Yes O No
   b. 30 days of admission (even if outside of your hospital)?
   O Yes O No

13. Do clinicians (e.g., physicians, physician assistants, nurses, etc.) from your hospital meet formally with emergency medical system (EMS) providers to review the care of patients with AMI?
   O Yes, about monthly
   O Yes, about quarterly
   O Yes, about annually
   O Yes, other ____________________
   O No

14. Does your hospital employ an EMS coordinator whose primary role is to help communicate with EMS agencies?
   O Yes O No

15. Does hospital staff provide formal training at least quarterly for EMS providers on the care of patients with AMI (for instance, teaching EMS continuing education classes)?
   O Yes O No

16. Does your hospital have a 'Morbidity and Mortality' (M&M) format to review and discuss every AMI patient who dies in-hospital or at 30 days?
   O Yes O No

17. Does your hospital use root cause analysis or a similar method to understand problems in AMI care?
   O Yes O No

18. Is your hospital part of a regional effort or consortium of hospitals to improve AMI care?
   O Yes O No

Note: Please reflect upon your hospital's quality improvement efforts that are **CURRENTLY** in place.

19. On the inpatient units, does your hospital have the following electronic capabilities?
    a. Computerized assisted physician order entry
        O Yes O No
    b. Computer prompts to alert user to potential drug-drug interactions or allergies
        O Yes O No
    c. Computer prompts to alert user to medication order expiration
        O Yes O No
    d. Computer prompts to improve adherence to core measures for AMI care (e.g., beta-blocker use)
        O Yes O No

20. Are there protocols used to guide nurses on when to call the attending cardiologist for patients with AMI?
    O Yes O No

21. Are nurses in any of your critical care areas cross-trained to cover in the catheterization laboratory?
    O Yes O No

22. Are patients with AMI typically assigned a designated staff member (e.g., case manager, discharge planner) within 24 hours of admission to arrange for post-discharge services?
    O Yes O No

23. Which of the following best describes the role of pharmacists in caring for patients with AMI?
    O Pharmacists round on all patients in the CCU
    O Pharmacists round on all patients with AMI (including patients not in a unit)
    O Pharmacists do not round, but review the medications of all patients with AMI
    O Pharmacists do not have a specific role in the care of patients with AMI
    O Other (please specify) ____

24. Does your hospital use standard protocols or processes of care for referring patients to cardiac rehabilitation?
    O Yes O No

25. Does your hospital use standard protocols or processes of care for referring patients to follow-up visits as an outpatient within 7 days?
    O Yes O No

Note: Please reflect upon your hospital's quality improvement efforts that are **CURRENTLY** in place.

Part II: Organizational Dynamics

In responding to the following statements, please draw on your own experiences in your current role working with clinical staff and administration to improve AMI care. Please rate the following statements in terms of how frequently you observe these occurring at your hospital.

26. Senior management uses data about AMI quality of care to make decisions (e.g., strategic, human resources, and compensation).
    O Never O Rarely O Sometimes O Usually O Always

27. Clinicians (e.g., physicians, physician assistants, nurses, etc.) caring for patients with AMI share evidence-based approaches with the AMI team.
    O Never O Rarely O Sometimes O Usually O Always

28. There is good coordination among the different clinical units involved with the care of patients with AMI.
    O Never O Rarely O Sometimes O Usually O Always

29. Emergency medicine physicians are engaged in efforts to improve AMI care.
    O Never O Rarely O Sometimes O Usually O Always

30. Emergency Medical Services (EMS) providers are valued members of the clinical care team for patients with AMI.
    O Never O Rarely O Sometimes O Usually O Always

31. Clinical units caring for patients with AMI (e.g., cardiology, emergency medicine) communicate effectively with each other.
    O Never O Rarely O Sometimes O Usually O Always

32. Clinicians (e.g., physicians, physician assistants, nurses, etc.) are encouraged to creatively solve problems related to AMI care processes.
    O Never O Rarely O Sometimes O Usually O Always

33. After we make changes to improve AMI care, we evaluate their effectiveness.
    O Never O Rarely O Sometimes O Usually O Always

34. Medical errors have led to positive changes in AMI care processes at the hospital.
    O Never O Rarely O Sometimes O Usually O Always

Please indicate your level of agreement with the following statements:

35. When changes are made, the reasons why are made clear.
    O Strongly Disagree O Disagree O Neutral O Agree O Strongly Agree

36. This hospital values the ideas of staff at every level.
    O Strongly Disagree O Disagree O Neutral O Agree O Strongly Agree

Note: Please reflect upon your hospital's quality improvement efforts that are **CURRENTLY** in place.

37. Staff feel that their suggestions will be taken seriously by senior leadership.
    O Strongly Disagree O Disagree O Neutral O Agree O Strongly Agree

38. Staff feel safe to speak up when they see gaps in quality or safety of care.
    O Strongly Disagree O Disagree O Neutral O Agree O Strongly Agree

39. Senior management holds staff members accountable for achieving results.
    O Strongly Disagree O Disagree O Neutral O Agree O Strongly Agree

Opinion leaders for AMI care in your hospital:

(Note: Opinion leaders can be defined as individuals perceived as having particular influence on the beliefs and actions of their colleagues)

40. Believe that our current practices for patients with AMI can be improved.
    O Strongly Disagree O Disagree O Neutral O Agree O Strongly Agree

41. Encourage and support changes in practices to improve AMI care.
    O Strongly Disagree O Disagree O Neutral O Agree O Strongly Agree

42. Believe that improving 30-day risk-standardized mortality rates after AMI is currently a priority in our hospital.
    O Strongly Disagree O Disagree O Neutral O Agree O Strongly Agree

43. Work cooperatively with senior management to make appropriate changes.
    O Strongly Disagree O Disagree O Neutral O Agree O Strongly Agree

In general, when there is agreement that change needs to happen:

44. Staff who care for people with AMI have the necessary support in terms of budget or financial resources.
    O Strongly Disagree O Disagree O Neutral O Agree O Strongly Agree

45. Staff who care for people with AMI have the necessary support in terms of training.
    O Strongly Disagree O Disagree O Neutral O Agree O Strongly Agree

46. Clinical units caring for patients with AMI have the necessary support in terms of facilities.
    O Strongly Disagree O Disagree O Neutral O Agree O Strongly Agree

47. Clinical units caring for patients with AMI have the necessary support in terms of staffing.
    O Strongly Disagree O Disagree O Neutral O Agree O Strongly Agree

Please indicate your level of agreement with the following statements:

48. If you make a mistake in this hospital, it tends to be held against you.
    O Strongly Disagree O Disagree O Neutral O Agree O Strongly Agree

Note: Please reflect upon your hospital's quality improvement efforts that are **CURRENTLY** in place.

49. In this hospital, it is easy to speak up about what is on your mind.
    O Strongly Disagree O Disagree O Neutral O Agree O Strongly Agree

50. The people in this hospital value others' unique skills and talents.
    O Strongly Disagree O Disagree O Neutral O Agree O Strongly Agree

51. Staff in the hospital are able to bring up problems and tough issues to the senior management.
    O Strongly Disagree O Disagree O Neutral O Agree O Strongly Agree

52. Our hospital has staff devoted to learning about the latest developments in AMI care and teaching others in the hospital about this new information.
    O Strongly Disagree O Disagree O Neutral O Agree O Strongly Agree

53. Staff within this hospital regularly share practical experiences with each other on how to improve AMI care.
    O Strongly Disagree O Disagree O Neutral O Agree O Strongly Agree

54. Our hospital has frequent interactions with outside organizations (e.g., other hospitals and professional associations) to acquire new knowledge on how to improve AMI care.
    O Strongly Disagree O Disagree O Neutral O Agree O Strongly Agree

55. Staff in different clinical units caring for patients with AMI regularly consult with each other on how to improve AMI care.
    O Strongly Disagree O Disagree O Neutral O Agree O Strongly Agree

56. Our hospital has difficulty implementing new initiatives to improve patient care.
    O Strongly Disagree O Disagree O Neutral O Agree O Strongly Agree

57. In this hospital, employees from different clinical units feel comfortable calling each other when the need arises.
    O Strongly Disagree O Disagree O Neutral O Agree O Strongly Agree

58. Efforts to improve care transitions for patients hospitalized with heart failure and AMI also have positive effects for transitions for other patients, such as those with pneumonia.
    O Strongly Disagree O Disagree O Neutral O Agree O Strongly Agree

Note: Please reflect upon your hospital's quality improvement efforts that are **CURRENTLY** in place.

59. We are trying to better understand your professional network. Please list the **hospitals** that you regularly interact with about efforts to improve the quality of cardiology care.

**Hospital Name**	**City**	**State**	How frequently have **you** provided **them** with information or tools to improve cardiology care?<br><br>1 = Once or twice a year<br>2 = Once or twice a month<br>3 = Once or twice a week	How frequently have **they** provided **you** with information or tools to improve cardiology care?<br><br>1 = Once or twice a year<br>2 = Once or twice a month<br>3 = Once or twice a week

60. Please list any other organizations (e.g., EMS agencies, professional associations) that you regularly interact with about efforts to improve the quality of cardiology care.

**Organization Name**	How frequently have **they** provided **you** with information or tools to improve cardiology care?<br><br>1 = Once or twice a year<br>2 = Once or twice a month<br>3 = Once or twice a week

Thank you for completing this survey.
We appreciate your time and interest.
